# Strengthening maternal nutrition counselling in the Philippines: reflections on recent evidence from Ethiopia

**DOI:** 10.1017/S1368980026102705

**Published:** 2026-05-22

**Authors:** Jessabell Cabada

**Affiliations:** Food Technology Department, College of Technologies, Bukidnon State University, Malaybalay City, Philippines

**Keywords:** Maternal nutrition, Nutrition education, Iron–folic acid supplementation, Antenatal care

Dear Editor,

The recently published article by Mohammedsanni *et al.* ^(1)^ provides compelling evidence that structured, facility-based nutrition education significantly improves dietary diversity and iron–folic acid supplementation among pregnant women in Ethiopia. Their cluster-randomised controlled trial demonstrates that repeated, standardised counselling supported by visual aids and delivered by trained health workers can meaningfully influence maternal nutritional behaviours in low- and middle-income settings. The relevance of these findings extends to the Philippines, where maternal undernutrition and micronutrient deficiencies remain persistent challenges. Goyena *et al.* ^(2)^ found that Filipino pregnant women had low consumption of nutrient-dense foods, including meat (42 %), dairy (31·9 %), eggs (30·8 %) and fruits (25·3 %) and were severely deficient in iron and vitamin A. These dietary patterns parallel those described in Ethiopia, underscoring the potential value of strengthened nutrition counselling within routine antenatal care. Global evidence reinforces this need. A 2024 systematic review and meta-analysis of fifty-three studies involving 13 475 participants reported that nutrition education increased iron–folic acid compliance nearly threefold (OR = 2·80), raised Hb levels by 0·88 g/dl and reduced anaemia risk by 34 %^(3)^. These findings align with the positive outcomes reported by Mohammedsanni *et al.* ^(1)^, collectively demonstrating that structured education is a consistently effective intervention across diverse low- and middle-income country.

However, contextual considerations are essential for successful adaptation to the Philippine setting. First, improvements in dairy intake and dietary diversity observed in Ethiopia may be constrained locally by food affordability and accessibility. Nutrition counselling must integrate practical, culturally relevant food options such as malunggay, small fish, root crops and fortified staples, which are also locally available and accessible. King’s field-based research^(4)^ further shows that Filipino mothers often have limited access to nutrition services and inconsistent quality of counselling. Second, antenatal care dropout rates in the Philippines are higher than in Ethiopia. Transport barriers, competing responsibilities and clinic congestion hinder repeated attendance, which is an important determinant of counselling effectiveness. Alternative delivery platforms, including barangay health stations, community health workers and digital tools, may help sustain exposure to the intervention. Third, inconsistent iron–folic acid supplement availability challenges adherence. The WHO’s MNCAH programme^(5)^ review guide emphasises that high-quality antenatal care requires standardised counselling and reliable commodity security to ensure supplementation continuity.

In light of these considerations, the Ethiopian model offers valuable lessons: structured, repeated and culturally adapted counselling that is supported by trained personnel and reliable supply systems could substantially strengthen maternal nutrition services in the Philippines.
